# Urban health indicators and indices—current status

**DOI:** 10.1186/s12889-015-1827-x

**Published:** 2015-05-16

**Authors:** Richard Rothenberg, Christine Stauber, Scott Weaver, Dajun Dai, Amit Prasad, Megumi Kano

**Affiliations:** School of Public Health, Georgia State University, Atlanta, GA USA; Department of Geosciences, College of Arts and Sciences, Georgia State University, Atlanta, GA USA; The World Health Organization Center for Health Development (The WHO Kobe Center), Kobe, Japan

**Keywords:** Urban metrics, Health disparities, Indicators, Indices

## Abstract

Though numbers alone may be insufficient to capture the nuances of population health, they provide a common language of appraisal and furnish clear evidence of disparities and inequalities. Over the past 30 years, facilitated by high speed computing and electronics, considerable investment has been made in the collection and analysis of urban health indicators, environmental indicators, and methods for their amalgamation. Much of this work has been characterized by a perceived need for a standard set of indicators. We used publication databases (*e.g.* Medline) and web searches to identify compilations of health indicators and health metrics. We found 14 long-term large-area compilations of health indicators and determinants and seven compilations of environmental health indicators, comprising hundreds of metrics. Despite the plethora of indicators, these compilations have striking similarities in the domains from which the indicators are drawn—an unappreciated concordance among the major collections. Research with these databases and other sources has produced a small number of composite indices, and a number of methods for the amalgamation of indicators and the demonstration of disparities. These indices have been primarily used for large-area (nation, region, state) comparisons, with both developing and developed countries, often for purposes of ranking. Small area indices have been less explored, in part perhaps because of the vagaries of data availability, and because idiosyncratic local conditions require flexible approaches as opposed to a fixed format. One result has been advances in the ability to compare large areas, but with a concomitant deficiency in tools for public health workers to assess the status of local health and health disparities. Large area assessments are important, but the need for small area action requires a greater focus on local information and analysis, emphasizing method over prespecified content.

## Introduction

“When we look at health problems on a world scale, we see bewildering diversity.” John Bryant’s classic 1969 work, *Health and the Developing World*, [1] begins with a dictum no less true today. Early in the book, he cites a composite index of human resource development based entirely on two measures of education (enrollment in the second level of education plus enrollment at the third level of education times five [2]), and stresses that “no weight should be put on the precise location of any one country in this ranking.” Thus, nearly 50 years ago, some of the chief problems with indicators and indices were well understood.

Against a backdrop of chaos and development, improvement in data systems and technology made health data more available in the ensuing decades, but the problems of summarization and interpretation persist. Scale is one of the critical factors in developing indicators and indices. The type and number of indicators, how they are presented, transformed and combined, the size of the targeted area, the relative placement of geographic units—all are scalable factors in the construction of an overall assessment. Indeed, the audience for the assessment is also scalable—from neighborhood groups to global agencies. Issues of scale, and the tension between multiple indicators and single statistics, suggest the need for a variety of alternative approaches.

A recent compendium of composite measures of human progress provides an exhaustive listing of extant indices from many areas of human endeavor [3]. This review, whose content overlaps in part with that compendium, will focus on indicators and indices that are relevant to urban health and urban health disparities in countries with advanced economies as well as low and middle income countries (LMICs). The emphasis will be on the types of urban metrics that are extant, the measures and methodologies used to assess health disparities, the comparability of these measures, and the extent to which single (vs. multiple vs. parsimonious) measures have been used to assess urban health and health disparities. Though of substantial importance in the construction of metrics, the statistical methodology has been discussed and reviewed in detail, and will not be a major focus here. Nor will specific disparities be featured. But in light of the urban orientation of the review, measures of health and environment will be paramount, together with mechanisms that have been used to amalgamate them.

### Measurement of health and disparities

**Fig. 1:**
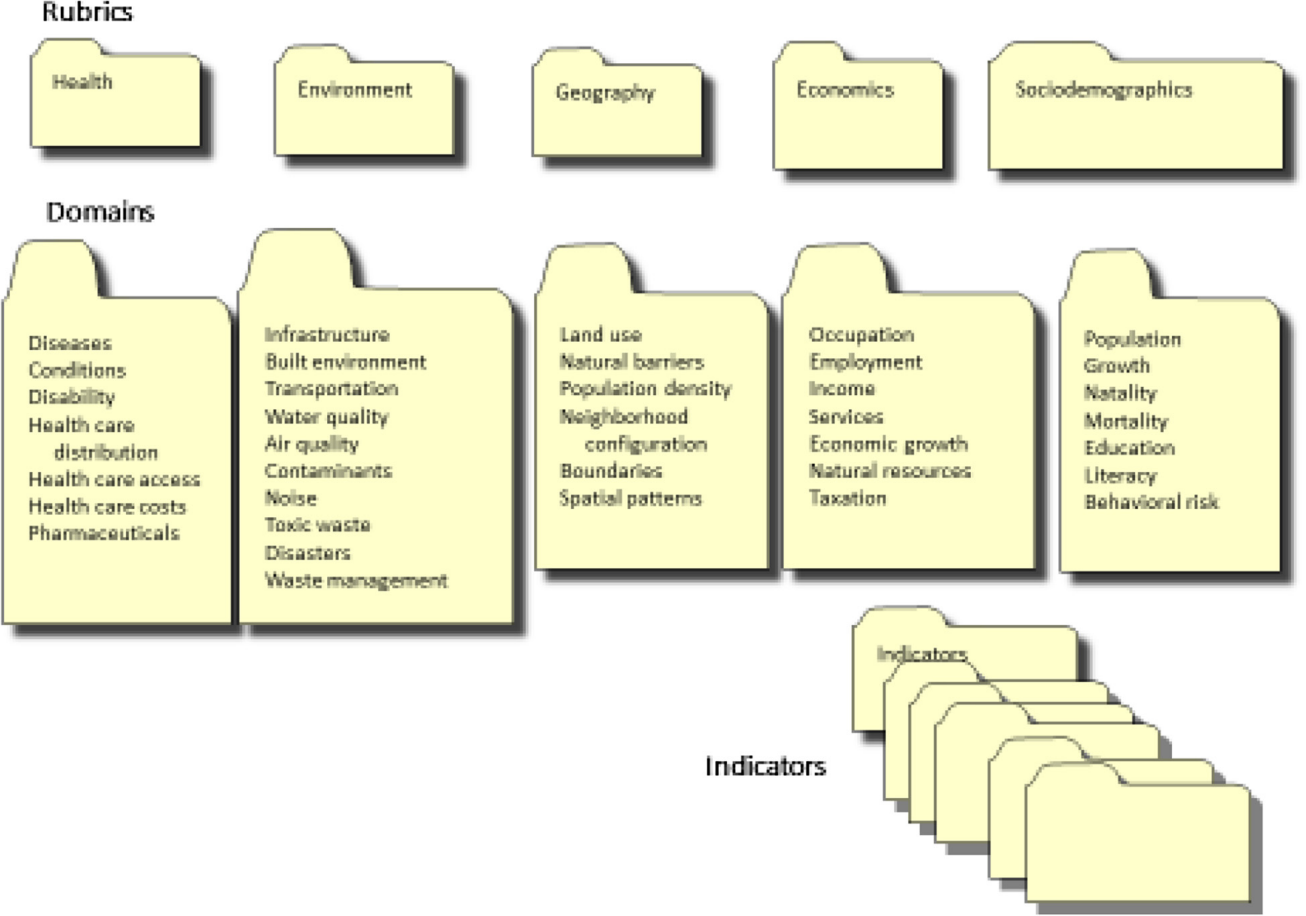
Hierarchy and nosology of terms used to describe measures of health and health disparities

A convenient framework for classifying the available measures establishes three levels of measurement: Rubrics, Domains, and Indicators (Fig. 1). The descriptive names used here—there are many valid alternatives—are a convenience for stressing the distinction among logical types. Rubrics represent societal-level factors that affect health, either directly, or as determinants. Domains are specific factors within a Rubric for which measurements are available. For example, the Rubric “Environment” includes the Domain “Air Quality” that contains a set of Indicators (e.g. “Proportion of households living within 300 m of major industrial stationary sources of air pollution”) from which potential disparities can be derived. To complete the vocabulary, for this review we will use “Index” to refer to a single measure, figure or picture that is constructed from Indicators. The term “metric” is used generically to refer to any measurement.

#### General properties of indicators

Soon after the Millennium Declaration, a Health Metrics Network, [4] funded by the Gates Foundation, was established to assist member nations in developing and interpreting health data. This partnership has recognized the key relationship between an indicator of health and an indicator of health disparity, and has provided leadership in deriving the latter from the former. An important contribution of those involved in the Network is a concise summary of the methodologies available to examine indicators and assess inequalities (first published in Spanish [5] and subsequently in English; [6–8] this discussion was based on Mackenbach and Kunst; [9] see also Houweling et al. [10]).

The authors point out that the vast majority of Indicators are based on data aggregated by geopolitical unit. A subset are cumulative markers (Gross National Product (GNP), percent of literacy, unemployment rate) that lack meaning at the individual level [6]. It is readily inferred that most analysis of Indicators is ecological, and thus constrained by the statistical limitations of correlational analysis. For example, in constructing an Index, indicators that co-vary do not necessarily increase the amount of information in the Index (though in some instances, such as a latent variable construct, they may). But conversely, indicators that are not correlated render interpretation of the Index problematic, since the level and trajectory of the Index then results from the complex interaction of disparate measures.

#### Capacity for demonstrating disparity

Perhaps more important, the authors [6] point out that indicator selection should be predicated on the ability to demonstrate disparity. By using information on the total population, and being sensitive to the size and distribution of the population along socioeconomic groupings, an overall indicator (say, GNP) can be transformed into a measure of inequality. In these articles, the authors then describe the major ways in which disparities are expressed (see Table 1). In fact, all of these are some form of ratio or difference, manipulated to highlight certain aspects of the contrast. For example, the measures that are based on the slope of a regression line (the ratio of a change in a variable compared to a unit change in another) simply provide a model-based contrast as opposed to the simple empirical observation of say, the ratio of highest percentile to lowest. The Lorenz Curve and the Concentration Curve are more complicated ratio measures, and have been shown to be specific examples of the class known as Relative Distribution Measures. Such measures raise a fundamental question for all methods of representing disparities. A simple ratio is dimensionless, and can thus be used for the comparison of many populations. A simple difference is in units of the underlying measures (money, frequency, incidence, area, etc.); some of these units permit direct comparability and others do not. Other measures of disparity compare observed data to an absolute standard (such as one of no disparity, as with the Lorenz curve and Gini coefficient). Still others use a standard embedded in the total data (say, highest or mean value). Relative distribution measures, [11, 12] on the other hand, compare two distributions directly so that, for example, the level of disparity in two urban areas can be directly described. Other measures permit this, but may require extra steps. In addition, the common practice of rank ordering, if performed without reporting the actual disparities, would not be sufficient to provide the actual difference between two areas since the space between ranks is not uniform.

**Table 1:** Summary of major methods for measuring inequality [9–16]

Measure	Definition
Ratio	The quotient of rates or values
Difference	The difference of rates or values
Effect index	A regression slope
Population Attributable Risk	A given rate compared to some baseline rate
Index of dissimilarity	Percent of cases that would have to be redistributed to have the same rate for all SES groups
Slope Index of Inequality	Slope of the regression line of a health measure against rank ordered SES category
Relative Index of Inequality	Slope Index of Inequality divided either by the mean or the highest level of the health measure
Lorenz Curve	Cumulative proportion of the population plotted against cumulative proportion of a health variable; the 45^o^ line represents uniform distribution
Gini Coefficient	Twice the area between the empirical Lorenz curve and its diagonal, a summary measure of deviation that corresponds to the amount of inequality
Concentration Curve and Concentration Index	Similar to Lorenz curve and Gini coefficient, but health variable is plotted against ordered socioeconomic status
Relative Distribution Measures	A more general class of measures that permits direct comparison of two distributions
Symmetrized Renyi Index	A measure based on entropy that is invariant with respect to a reference group and that permits judgment-weighting based on the perceived importance of disparities

The references cited in the Table heading include several summary articles on these measures, and an extensive discussion of Relative Distribution Measures

More recently, Talih [13] introduced the symmetrized Renyi Index, based on prior work using entropy measures to assess disparities. An important advantage of this measure is its invariance with respect to a reference group (say, the population average or the least well off group). In addition, population-weighted and equal-weighted versions can be calculated, and an “aversion” parameter can be included that reflects the investigator’s judgment as to values that society attributes to inequality [14].

#### Criteria for indicators

The choice of indicators, from the myriad available, should be predicated on some agreed upon set of criteria. Flowers et al. [15] provide a checklist of 20 facets of a proposed indicator: several are descriptive (title, origin, rationale, routine or special collection, frequency); others deal with general characteristics (strengths, weaknesses, perverse incentives, influence on practice or behavior). A simpler, and perhaps more forceful summary of the ideal characteristics of indicators is provided by Etches et al. [16] whose keywords bear repeating: consensual, conceptual, valid, sensitive, specific, feasible, reliable, sustainable, understandable, timely, comparable, and flexible. These authors stress as well the need for a conceptual framework [16], Fig. 1, p.34 from which the appropriate indicators can be drawn and indices can be constructed. Such a framework can be the basis for multilevel modeling [17] and for causal analysis [18]. Though neither of these approaches is necessarily involved in the formation of indices, they are part of the intellectual basis for prior and subsequent analysis.

#### Pitfalls and problems

Several statistical problems bedevil indicators. The Will Rogers phenomenon, for example, is the paradox observed “when moving an item from one set to another moves the average values of both sets in the same direction,” [15], p.243 and refers to migration of an item to a group vastly different from its own. Such a situation obtains when the highest value in one population is less than the lowest value in another, so that movement of the highest item lowers the mean of both groups. Indicators are also subject to regression to the mean, a phenomenon that reflects the random distribution of measurement error. A more extreme value will likely be followed by one less extreme because, based on typical distributions, the error in measurement of the second value is likely to be less extreme than that of the first value. As noted earlier, indicators or indices are often presented as ranks, which are ordinal rather than interval or ratio quantities despite the use of integers. Ranks convey a sense of better or worse that may not be merited by the underlying data. In addition, entities are not equally separated, and some may be bunched so that ties are resolved by resorting to a non-meaningful number of significant digits. Most indicator assessments and ranking procedures do not contain an appropriate estimate of uncertainty, and an assumed difference may be spurious. Flowers et al. [15] suggest the use of such devices as funnel plots (a standard part of the meta-analysis armamentarium) to detect aberrations in the distribution of values that may point to real differences.

#### Aggregation of indicators

As described by Saltelli et al. [19] indices are composite statistics that have generated polarized views of their value: either a mashed together collection of unrelated numbers or a usable distillation of reality. But these authors go on to point out that such statistics are really mathematical models developed through a social process: the community of scientists, policy makers, and practitioners must largely agree on their makeup and utility. The European Commission Joint Research Centre group on Composite Indicators [20] has explored the mathematical, political, social, and economic aspects of composite indicators in detail [19, 21–26]. This complex analysis provides a rigorous basis for combining criteria and for legitimate ranking schemes. As these and other investigators point out, linear aggregation, with either equal weighting or some other weighting scheme, is simplest, most commonly used, and often least reproducible, in that it does not derive from pre-established criteria, but rather from experience and negotiation. Geometric aggregation, usually by multiplying the *n*th root of *n* items, has been used successfully by the UNDP Human Development Index [27] but does require higher technical capacity. The most complicated of the approaches—multi-criteria analysis [28, 29] —is less adaptable for use on the local level, but a toolbox of techniques has been developed, [21, 22] and the use of this approach is a good example of the potential value of an academic and public health partnership [30].

### The major compilations of indicators

The current large collections of indicators differ substantially in genesis and purpose (Table 2). WHO’s Urban HEART, [31] the Michigan Critical Health Indicators, [32] and San Francisco’s Healthy Development *Measurement* Tool (now renamed the Sustainable Communities Index) [33] were all constructed, in part, to permit local areas to assemble and assess their own data. The United States’ Healthy People 2020 [34] was constructed as a mechanism for tracking progress toward national health goals, and focuses predominantly on individual risk. The Community Health Status Indicators [35] are an interactive tool for localities to assess their situation. Cities Environment Reports on the Internet (CEROI), [36] the CDC’s Environmental Health Indicators, [37] and California’s Environmental Health Indicators [38] focus primarily on environmental measures, many of which are urban. Women’s Health Indicators [39] are a compendium from many sources whose focus is how the indicators apply to women. Similarly, UNICEF’s compilation applies to children, and is a tool for tracking Millennium Developmental Goals [40]. The WHO Indicator Compendium [41] (on a large scale), and the Social Health of the States [42] (on a smaller scale), are general sets of measures that includes elements of both personal and environmental health. The World Bank’s World Development Indicators [43] is primarily economic and political in orientation but has considerable information on health and urban development. Global Cities Indicators, a set of measures on 20 themes that measure city services and quality of life, have been developed by the Global Cities Institute of the University of Toronto, and is available to member cities only [44].

**Table 2:** Multiple indicator compilations

Compilation	Description
WHO: Urban Health Equity Assessment and Response Tool (Urban HEART) [31]	http://www.who.int/kobe_centre/measuring/urbanheart/en/index.html
	This long term project of the WHO Kobe Center and its collaborators provides member states with a tool to assess inequalities in health status in their urban centers. In addition to a core set of health care outcomes (number of indicators [4] and health determinants [8]), it has a set of strongly recommended outcomes (4), physical environment and infrastructure variables (3), social and human development measures (6), economic indicators (3) and governance indicators (2). Since its primary focus is urban, it includes variables not found in many of the other projects (e.g. households served by solid waste management systems; solid fuel use; improved sanitation).
Healthy People 2020 (US Department of Health and Human Services) [34]	http://www.healthypeople.gov/2020/default.aspx
	The Healthy People initiative is a 30-year project, updated every 10 years that attempts to track the progress made in population health in the United States. It compares currently available data to a set of predetermined goals to judge that progress. The 26 leading health indicators within 12 topic areas focus on personal behaviors, environmental quality and access to health care. The major effort here is to provide an agenda for prevention, rather than a metric, so that HP2010 is not as germane to the current task, but does provide an exhaustive list of indicators. An historical overview and comprehensive summary of current indicators is found in:
	http://www.healthypeople.gov/2020/about/history.aspx
	A current update may be found at: http://www.healthypeople.gov/2020/leading-health-indicators/Healthy-People-2020-Leading-Health-Indicators%3A-Progress-Update
WHO Health Compendium Indicators, 2012 [41]	http://www.who.int/gho/publications/world_health_statistics/WHS2012_IndicatorCompendium.pdf
	The Health Compendium Indicators provide the metadata for an array of health indicators that are used in many contexts by WHO. As titled, it is a registry of data and sources, and does not in itself purport to be a set of metrics or indicators. It does, however, provide a wide array of measures that can be incorporated into an urban health metric.
Michigan Critical Health Indicators (Michigan Department of Community Health, USA) [32]	http://blogpublic.lib.msu.edu/index.php/state_of_michigan_cities_an_index_of_urb?blog=5
	This set of indicators was designed to measure the health and health behaviors of Michigan residents. The current report (2011), which is organized by 4 specific health topics and their 28 related measures or indicators, is upbeat. It asserts that, in general, the health of Michigan’s population is improving, with only a few indicators going in the wrong direction (adult obesity, diabetes, chlamydial infection), and a number of health disparities were documented. The report is a good demonstration of the use of health metrics in the longitudinal analysis of population health trends. It does not provide a single, or composite metric, and is not focused exclusively on urban areas.
Community Health Status Indicators (US Department of Health and Human Services) [35]	http://wwwn.cdc.gov/CommunityHealth/homepage.aspx?j=1
	The CHSI provides an online interactive site for United States counties to get information about themselves. It includes demographics, summary health measures (life expectancy, all-cause mortality, self-rated health status, and average number of unhealthy days), leading causes of death, vital statistics, environmental health, preventive services, risk factors, and access to care. It provides a county with the data elements compared to the US national average, dividing the results in 4 quadrants, so that a county can see for which measures it is doing better or worse than the national average. This is an interesting approach that provides an overview to a small population unit of how it is doing compared to everyone else. It is not specifically urban, and does not provide a single or composite measure, but is a useful approach and reflects the techniques used by a number of international sites.
Environmental Health Indicators (CDC, USA) [37]	http://ephtracking.cdc.gov/showIndicatorsData.action
	The US CDC maintains a site for Environmental Public Health Indicators (EPHI), with a larger set of metrics, and a smaller set of core indicators. The purpose is to provide a framework for state and local health departments to make a comprehensive assessment of environmental hazards. The actual measurements, and their analysis and synthesis, are to be obtained by the agency using the framework. In this regard then, the site provides a useful set of metrics, but does not pursue data collection, analysis, or judgments itself.
Environmental Health Indicators (California, USA) [38]	http://www.ehib.org/papers/health_indicators.pdf
	The California Department of Health Services, Environmental Health Investigations Branch, compiled a set of 18 indicators that overlap to a large extent with Healthy People 2010 and with the WHO frameworks. The list focuses on environmental hazards, but includes measures on population, demographics, health, health outcomes, and some specific measures related to California. It provides little that is new or original, but the discussion of each measure provides a good summary for the state.
National Women’s Health Indicators Database (U.S. Department of Health and Human Services) [39]	(http://www.healthstatus2020.com/index.html)
	The site uses measures from a variety of other efforts. It is an assemblage of metrics that relate primarily to women’s health, but it does not break new ground. The measures are derived from other sites.
European Urban Health Indicators System [75]	http://www.urhis.eu/
	This project is an ongoing development of a set of comparable urban health indicators in 60 urban areas of Europe. It uses a newly developed health survey instrument and other routinely available data (such as mortality statistics). It focuses specifically on urban area data to “provide tools for evidence based policy.” It is a work in progress, and will have as its product a wide array of tools and metrics for cross sectional and longitudinal assessment. The project does not attempt to provide a single urban metric or composite statistic, but rather to provide a basis for ongoing analysis and decision making with complex data. Its emphasis on the development of local perspective creates important similarities with Urban HEART. Euro-URHIS 2, recently completed, has begun publication of its findings.
Sustainable Communities Index (San Francisco, USA) [33]	(http://www.sustainablecommunitiesindex.org/)
	The San Francisco Department of Health has created a website that can be used as a workbook for assessing local health and environment status. This website provides a sophisticated and comprehensive workbook of all the major items related to urban health and environment. Neighborhoods of metrics available through the Census and through other public sources). This grouping attempts to connect public health concerns to urban development planning. There are two primary indicators of Health Systems along with numerous others grouped under Environment, Transportation, Community Cohesion, Public Realm, Education, Housing and Economy.
Cities Environment Report on the Internet (CEROI) [36]	www.unep.org/ieacp/files/pdf/Geo_Cities_Manual_ECCA.pdf
	CEROI is a Norwegian-based organization tasked with providing cities sound environmental information for decision making. To that end, it prepared a common set of 90 indicators. These are organized in a set of 29 core areas, and was built on prior efforts by a number of European entities: European Common Indicators; European Environmental Agency indicators; European Foundation for the Improvement of Living and Working conditions; International Council for Local Environmental Initiatives; and United National Center for Human Settlements. Both the focal areas and the list of indicators are concordant with many of the other major lists already cited.
UN HABITAT : Habitat Agenda Urban Indicators [76]	http://ww2.unhabitat.org/programmes/guo/documents/urban_indicators_guidelines.pdf
	A part of the Millennium Developmental Goals (MDG), the UN HABITAT developed a set of Habitat Agenda Urban Indicators: 20 key indicators; 9 check lists; and 13 extensive indicators. For ease of data collections, they are grouped into two clusters: those obtained from censuses, household surveys, DHS surveys and Multiple Indicators Cluster Surveys (see below); and those from other sources, including official records, housing boards, financial institutions, police, NGOs, and informed estimates. This is targeted specifically to cities, and provides direct instruction to localities on how to collect information to serve the 8 MDG areas. The listing itself is a comprehensive look at the built environment, but also includes several measures on social development, environmental management, economic development and governance.
World Development Indicators [43]	http://data.worldbank.org/products/wdi
	The World Bank has developed a set of 508 indicators covering 217 countries for the period from 1960 to 2013. These 50 years are a treasure trove of economic data, but there are as well a set of 36 health indicators and 17 urban indicators. It appears that no attempt has been made at further amalgamation of these variables, but their high concordance with other groupings, and the breadth and depth of the data make them an invaluable resource.
UNICEF: Data: Monitoring the Situation of Children and Women [40]	http://data.unicef.org/index.php?section=unicef_aboutus
	UNICEF supports countries in collecting data related to children and women through Multiple Indicator Cluster Surveys (MICS), currently in its fifth iteration. The wealth of data available on children and women’s health make this a valuable source for construction of an index, though that does not appear to be part of the overall agenda of this activity. Many of the variables related to children and attendant issues may be found in the other major projects as well.

With such diversity of purpose, it is no surprise that there is little concordance in the naming of Rubrics, Domains, or Indicators, or in the number of indicators. World Development Indicators, for example, has collected a set of 508 indicators on 217 countries for the period 1960 to 2013. Seventy-six of these relate directly to health and the urban environment. At the other end of the spectrum, the UN Habitat Agenda Indicators number 26, and provide a good example of the type of informed choices that are made. Under a Domain heading that they call “Social Development and the Eradication of Poverty,” they choose six Indicators in order to capture the essence of the Domain: Under-5 mortality, Homicides, HIV prevalence, Literacy rates, School enrollment, and Women Councilors. In a similar vein, the WHO Kobe Center’s Urban HEART lists 12 “core” indicators, and 18 “strongly recommended” measures. Its rough analogy to the UN Habitat Agenda Indicators is a Domain called “Core indicators: health determinants” that contains: access to safe water; access to improved sanitation; completion of primary education; skilled birth attendance; fully immunized children; prevalence of tobacco smoking; unemployment; and government spending on health. Both lists of indicators are worthy, but they clearly take different routes to a similar goal. A cursory look at the remaining Indicator projects reinforces the sense of plethora rather than parsimony. But a more detailed look suggests a somewhat different picture. If similar or identical Domains in each major compilation are given a common name, a pattern of concordance emerges. Nine Domains appear in more than half of the aggregations, and three of them (health care, infant mortality, and education) appear in more than two-thirds. The qualitative impression is that there is a vast array of specific indicators, with little commonality among projects, but a relatively limited number of Domains that appear in many, if not most projects. These Domains deal largely with health care outcomes, though several social determinants of health (for example, education, poverty and environment) are represented as well. Thus, despite disagreement about detail, there is some evidence of agreement about basic content. This observation augurs well for the construction of more flexible indices that permit interchangeability of indicators.

### The properties of indices

There are only a few indices that are specifically urban in orientation, but a substantial number of congeners have been developed for other purposes. Consideration of the range of indices provides some insight into the appropriate methodologies for construction and validation (Table 3).

**Table 3:** Recent examples of Indices

Aggregation	Index Methodology
Social Health of the States (2008) [42]	http://iisp.vassar.edu/SocialHealthofStates.pdf
	The Institute for Innovation in Social Policy (Vassar College, Poughkeepsie, NY, USA) has a long running interest in tracking health and social equality in the United States. It uses 16 indicators, which are scaled so that the worst possible average is 50. The differences between a state’s actual average and the worst possible is expressed as a percentage of 50; the larger the percentage, the higher the social health score. States are ranked and the 50 states are grouped in quintiles, with ranks 1–10 deemed excellent, and ranks 41–50 deemed poor. Thus this project provides a set of indicators (see their table that is built on the life cycle, and has many points of concordance with those already discussed). In addition it calculates an Index from these that permits ranking and tracking of states. This is obviously not specific to urban areas, but is of interest in development of a metric for urban areas.
Michigan index of urban prosperity [45]	*www.landpolicy.msu.edu* (The initiative on Michigan prosperity is active, but the Index is not currently available, highlighting the evanescent nature of some of these enterprises.)
	This index, developed by the group that created the Michigan Critical Health Indicators (see above), combines multiple components: crime rate; property value change; median household income; employment rate; employment change; graduation rate; MEAP passing rate; young adults; population change. These indices are measurements that are compared to the overall state measurement, taken to be 1. They do not explicitly state how the 9 measures are combined to produce an overall index, though it would appear to be a simple average. They apply this index to a number of urban areas within the state, showing that Ann Arbor (a University town) is faring much better than its more gritty urban counterparts (such as Detroit). This effort is a good example of the attempt to construct an index (and could be classified as well as a Unitary Indicator), though it is not clear that it would be applicable in developing areas where much of the data might not be available. The initiative on Michigan prosperity is active, but the Index is not currently available, highlighting the evanexcent nature of some of these enterprises.
Index of Resident Economic Well Being [46]	In this older study, the authors developed a 5-component index that includes: unemployment rate; poverty rate; labour force participation; median household income; per capita income. These are combined using N-scores (like z-scores but use deviations from the median), but the details are not provided. It is another attempt to use a few indicators to form a unitary metric that can be used to compare areas.
	Noted also in their discussion: City Distress Index (city poverty rate, unemployment rate, per capita income growth, and population change); *James, F. (1990) City needs and distress in the United State : 1970s to the mid 1980s, in: M. Kaplan and F. James (Ed.) The Future of National Urban Policy. Durham, NC: Duke University Press*. Other measures are reviewed there as well, but all seem to follow a similar pattern. These metrics are specifically urban, but not specifically health-related.
UNDP: Human Development Index and new associated measures [27, 47, 77]	http://hdr.undp.org/en/statistics/hdi/
	http://hdr.undp.org/en/content/human-development-index-hdi
	The Human Development Index focuses on three fundamental measures: life expectancy at birth; mean years of schooling and expected years of school; gross national income per capita. Each of these measures is “normalized” by taking the country value as a percent of the range of the most extreme values ([country value – minimum value]/[maximum value – minimum value]). The two measures of education are then combined by taking their geometric mean, and this combined value is further combined with the other two measures using the geometric mean. The result is a value between 0 and 1.0 that reflects the relative place of each country in the overall ranking of nations. The measure is thus complex in its creation but simple in its interpretation. It serves as an interesting model for a possible Urban Health Index, which could be constructed from a small number of constantly recurring measures, or a possible Urban Health Disparities Index, with similar characteristics.
	The HDI has now been augmented by a number of similarly-constructed measures whose characteristics have considerable ramifications for the development of an Urban Health Index:
	Inequality-adjusted Human Development Index (http://hdr.undp.org/en/statistics/ihdi/)
	Gender Inequality Index (http://hdr.undp.org/en/statistics/gii/)
	Multidimensional Poverty Index (http://hdr.undp.org/en/statistics/mpi/)
	Each of these requires considerable mathematical manipulation and might not be readily accessible by health workers in country, but they provide a sophisticated example of an approach to a unitary index that may have value.
Bertelsmann Transformation Index [49]	http://www.bertelsmann-transformation-index.de/en/
	Though not directly related to health and urbanicity, the BTI is an interesting example of index construction by a somewhat different route. A total of 17 criteria subdivided into 52 “questions” are provided in a country report for each of the 129 participating nations. Experts on subject matter and from the participating countries review and “calibrate” the numbers which are then subjected to a final review by the BTI board. The scores are combined using linear aggregation (the exact method is not reported on the website) and an overall score for a number of domains is assigned. The major difference between this Index and many of the others is that it employs a modified Delphi technique rather than simply aggregating available statistics. An approach of this sort may be of value in developing an index, but might not be workable on the local level.
Corruption Perception Index [50]	http://www.transparency.org/policy_research/surveys_indices/cpi/2010
	Transparency International has created an index that ranks countries according to their perception of corruption in the public sector. It uses a minimum of three sources for each country, carried out by “independent and reputable institutions” (their web materials cite 12 different surveys from 11 listed organizations, of which the BTI, above, is one). The data are standardized using matching percentiles, then undergo beta transformation for which the cumulative distribution function is used. The final CPI is the linear average of the transformed values. This complex process, involving two important transformations is thus based on an amalgamation of impressions from many sources, and rests heavily on a Delphi process as well. The final product is a rank ordering of nations, with attendant political ramifications.

Not all of these are related directly to health, but they provide a sense of the spectrum of index construction that has been attempted in recent years

#### Simple indices

In its simplest form, an index is constructed from a set of indicators that have been transformed (standardized, normalized, scaled) so that they are directly comparable, and then added together. Simple arithmetic combination, often mistakenly called “unweighted,” implies that each indicator is given the same unit weight. The resulting Index may be bounded (such as a proportion or percent) or unbounded at one or both ends. A simple example is the Social Health of the States, [42] a long-running Index from the Institute for Innovation in Social Policy. It combines 16 indicators that have been scaled and averaged so that the worst possible score is 50 (smaller is better). The difference between a state’s actual average and 50 is then expressed as a percentage of 50. The states are rank-ordered and grouped in quintiles (1–10 are excellent; 41–50 are poor). Similarly, the Michigan Index of Urban Prosperity [45]—one of the specifically urban indices—combines nine indicators from multiple sources (crime rate; property value change; median household income; employment rate; employment change; graduation rate; Michigan Education Assessment Program passing rate; young adults; population change). It uses the ratio of each site-specific indicator to the overall state indicator (actually, to the overall mean) and averages them, deriving a number in the vicinity of 1.0. A somewhat more complicated urban metric is the Index of Resident Economic Well Being, [46] which combines indicators from five Domains (unemployment rate; poverty rate; labor force participation; median household income; per capita income) by using a linear combination of N-scores (deviations from the median, as opposed to z-scores, which are standard deviations from the mean).

#### More complex indices

Perhaps the most important of these is the Human Development Index (HDI) [27] now in its 25^th^ year, published by the United Nations Development Program (UNDP). The measure is constructed from life expectancy at birth, measures of schooling and expected years of schooling, and gross national income per capita. Each of these is standardized by taking the country value as a percent of the range of the most extreme values for any participating country over the past 20 years compared to subsistence value: ([country value – subsistence value]/[maximum value – subsistence value]). The resulting value is a proportion between 0.0 and 1.0. The two education values are combined by taking their arithmetic mean and combining them with the other two measures using their geometric mean ([life expectany^1/3^ x schooling^1/3^ x income^1/3^]). A country’s HDI is then rank-ordered among all the others, and its place over time can provide the size and direction of relative progress (“relative” because a country’s change in rank may not reflect its change in absolute values). Since the standards for “best” and the “worst” are fixed, and each nation’s values are placed on a scale with that range, the concept of disparity is an integral part of the measure. Though urbanicity is not the focus of the HDI, its approach and methodology are suited to the development of a measure of urban health and disparities. In addition, the UNDP introduced an inequality-adjusted HDI, [47] a measure that accounts for inequality by adjusting each indicator’s value by its level of inequality, based on work by Atkinson [48] wherein he used the analogy between ranking inequality distributions and ranking probability distributions based on utility. Each of the three indicators is adjusted by the ratio of the geometric mean of the distribution to its arithmetic mean. Using a similar statistical approach, they have also introduced a Gender Inequality Index that captures the difference in reproductive health, empowerment and the labor market for men and women. A third measure—the Multidimensional Poverty Index—diverges from the Human Development Index by using microdata from household surveys. Each person is classified as poor or non-poor based on his or her family deprivation and the data are aggregated to form a national index. The actual computation bears considerable resemblance to previously discussed aggregations of indicators (weighted linear combinations), though the mechanism for combining information on the 10 indicators used is complex.

Another example of a more complex measure, the Bertelsmann Transformation Index (BTI) [49] takes a wholly different approach. In their process, 17 criteria (“Domains”) are represented by 52 questions (“Indicators”) that are answered in a report completed by 128 participating nations. The answers go through two levels of review and calibration (not further defined) by experts in the responding country and by the BTI board. Scores are combined by linear aggregation (not further defined), and an overall score and sub-domain scores are calculated. The approach may be described as a modified, interactive, Delphi technique that is heavily dependent on expert opinion, and may or may not be reproducible. Such an approach, however, recognizes, and in fact embraces, the political process that is an important part of index development.

A third approach is typified by the Corruption Perception Index [50] produced by Transparency International that ranks countries by the perception of corruption in their public sector. They collect information from a variety of sources (of which the BTI is one) and use at least three different sources for each country. This approach represents a substantial divergence from most of the others in that a uniform data set is not used for each country. Rather, they subject available information to substantial mathematical manipulation: data are standardized by using matching percentiles (reminiscent of the relative distribution methods), then undergo beta transformation, and a linear average of the transformed values is taken. The final index and ranking are substantially removed from the raw information. This approach acknowledges presumed exchangeability of indicators after mathematical manipulation.

Still another approach might be termed the “organic” Index, one that grows, shape-shifts, and is tested for its credibility and consistency. An example is the Deprivation Index, first proposed by Townsend [51] in 1987 and Carstairs [52] in 1989. These were constructed as the sum of four standardized variables. The Townsend Deprivation Index used percentage of unemployed people in the active population, percentage of not-owner-occupied households, percentage of households without a car, and percentage of overcrowded households. The Carstairs Index replaced no-owner-occupied households with the percent of low social class persons (a measure available in England based largely on occupation). In a subsequent review of Deprivation Indices [53], Carstairs describes other variations, such as the Jarman Underprivileged Score [54], constructed from rankings by general practitioners and subsequently used as part of a reimbursement scheme. Carstairs demonstrates that the Deprivation Index, as she developed it, was strongly correlated with measures such as overall mortality and cancer registrations.

In more recent years, the Deprivation concept has been retained, but the details altered. Sivakuman [55] proposed a Human Deprivation Index based on percent below the poverty line, infant mortality, and illiteracy rate. One-third of each is added together to form the Index. Messer et al. [56] constructed a Deprivation Index based on five sociodemographic domains: income/poverty, education, employment, housing, and occupation. They used principal components analysis, taking the first principal component as representative of neighborhood deprivation, an assertion supported by the consistency of component loading across study areas. Rey et al. [57] explored the properties of their previously developed Index, FDep99, which had been constructed from: median household income; the percentage high school graduates in the population aged 15 years and older; the percentage blue collar workers in the active population; and the unemployment rate. This measure was also constructed using principal components analysis, and the first principal component accounted for 68 % of total variation in mortality. The authors provided an empirical analysis that purported to show that FDep99 was superior to their slightly altered versions of the Townsend and Carstairs Indices.

The aforementioned Indices are instructive in providing a typology, but only touch on the extant composites that have been developed. In a systematic review, Kaltenthaler and colleagues [58] described 18 health indices culled from the literature from 1966 to 2000, and summarized information on their origin (US, UK, Canada, and Europe), characteristics, purpose, types of indicators, methods of aggregation, data sources, and validation. Several major points emerged. First, only four of the indicators had been validated, two by professional judgment, two by inference. Second, the user groups were not clearly defined, so that the target geopolitical level was not always clear. Reasons for choice of indicator were opaque. Weights appeared to be arbitrary, or at least not justified by standard criteria. The data upon which many of these indices were based were not always publically or universally available. The authors concluded that this set of indices would not be suitable for health policy makers in the United Kingdom (the place of origin of the study). Nonetheless, the authors reaffirm “the need for a population-based health index at either national or local level.” [58], p. 254.

Unfortunately, the literature on Indices that reflect urban health specifically is sparse. Those that include both urbanicity and health tend to focus on the former. As an example, Shane and Graedel [59] propose a set of indicators that includes a measure each for air, water, soils, transportation, energy, resource use, population, urban ecology, livability, and general environmental management. They do not include health measures per se, but do use the Human Development Index as an environmental measure. Instead of a composite index, they propose a novel graphic: a triangle made of four layers (planning, waste, resource, human factors). Each of the 10 metrics is represented in the triangle by a grey scale corresponding to its adequacy (high, middle or low rating). The resultant “picture” can be compared to triangles from other areas, and can be used as a marker for evaluation over time.

An exercise in index construction was conducted by Stephens et al. [60] who used a workshop environment to build an Index of Deprivation that compared Accra, Ghana with Sao Paolo, Brazil. Interestingly, groups working on the two areas devolved on the same Domains (income, education of head of household, number of persons per room, sanitation, and safe water access), but had to use different Indicators within those Domains. The collected data produced an overall picture that concealed substantial differences between the two areas. Those differences were demonstrated, however, by a simple choropleth map comparing the two cities by using four levels of socio-environmental conditions. Nonetheless, the authors felt that the data and resulting indices did not fully capture the political and social complexity of the cities. They do, however, cite several positive policy changes that resulted from the exercise. An important message from the study is the need for greater flexibility in the choice of indicators that make up an Index, since their true function may be as a catalyst for local change.

We have recently published [61] an Urban Health Index (UHI) that focused more on method than content. Adopting approaches used for the Human Development Index, [27, 47] the UHI permits construction of a variety of composite indices related to urban health, urban health disparities, and health determinants, and is coupled to a technique for mapping that provides visual display of disparities for contiguous small areas. Indices are standardized by transforming the values for each small area into a proportion of the range for the overall location, and are then combined by taking their geometric mean. The method, still under empirical investigation, may be of use in demonstrating health disparities and the geographic distribution of inequalities. It is an example of the reorientation of composite indices from methods for ranking to flexible tools for use by local public health workers to assess health status, needs, and disparities. In addition, it highlights the need to collect data as an integral part of the construction of indices. Small area data—differing only in scale from the more routinely collected large area data—are critical for understanding the urban microenvironment.

### Measuring the urban environment

The urban milieu has produced its own set of indicators, many of which are tied to health determinants. They are in a separate sphere of research, however, largely because of a differing measurement methodology, but also because of the well-known complexities of associating specific environmental hazards to health [62]. Recently, researchers estimated that almost 25 % of all disease burden can be attributed to the environment. The burden is estimated to be even greater—34 %--in children under 15 years of age, and to be of far greater consequence in LMICs compared to more developed countries [63, 64]. There is a growing need to be able to measure and use indicators of environmental health since they are a crucial link in the data and decision-making process [65]^, Ch. 3^. The purpose of the indicators is to express linkage between an environmental condition and health effect relevant at the policy level which may then facilitate effective decision-making.

Two general types of environmental health indicators have been described: exposure-based indicators and effect-based indicators [65] ^Ch. 3^. Exposure-based indicators measure environmental exposures with established health effects such as particulate matter with respiratory disease. Effect-based indicators typically measure a health effect that is commonly associated with an environmental exposure: for example, diarrheal disease and drinking water quality. Corvalan and colleagues [65] have suggested that environmental indicators must meet a dual standard: to be scientifically valid, and politically relevant. The latter would include being related to conditions that can be changed, easy to understand, acceptable to all stakeholders, and temporally cogent.

A variety of frameworks has been developed to assist with indicator creation and use. The most commonly cited framework for environmental health indicators is the “Driving forces, Pressures, State, Exposures, Effects and Action” or DPSEEA framework [66]. While based in part on the simpler pressure-state-response framework, this modified version has expanded to include the role of driving forces which are thought to be the key components that push environmental processes forward. As presented by Briggs (Fig. 1 in his publication [66]) the framework can provide a guide for the development of appropriate environmental health indicators for a range of situations. It also provides a tool to consider the various levels of environmental health interventions and how they may have impact on the different components of the model as provided in the “Action” component of the framework.

Over the last thirty years multiple projects have been undertaken to develop environmental health indicators. A composite set of indicators has not been developed although as evidenced by the comparisons of the other indicator sets, often many sets of indicators overlap. Even where these indicators overlap, few have been specific to urban environments. Lawrence [67] reviewed the body of work on environmental health indicators (Table 4) with a specific emphasis on those that have focused on cities. Lawrence puts forward a new research agenda for urban health indicators. He suggests that researchers “use indicators to identify sets of contextually defined components of each human settlement and its neighborhoods.” He also recommends identifying comparable sets of indicators that are useful for comparison across different types of “human settlements.” Finally, he stresses the need for spatial and temporal measures at the local level.

**Table 4:** Environmental Health indicators in major projects

Name of Project or Initiative	Description of project	Description of Indicators
UN-Habitat Global Urban Indicators Database [76]	City level data and conditions of urban areas	20 key indicators, 9 check lists and 13 extensive indicators. See also Table 2.
ICLEI - Cities 21 Project [78]	Objectives include to help establish trends in urban environment	A set of 70 indicators have been proposed
UNEP/GRID – CEROI Project [36]	Goal to facilitate access to information to help with decision-making at city level	A list of 90 indicators
		See also Table 2
European Sustainable Cities Report (European Common Indicators) [79]	Monitoring tool to measure impacts of urban activities for local and regional authorities	10 sustainability indicators
UK Sustainable Development Indicators [80]	Proposed to monitor sustainability and quality of life	12 headline indicators and 23 supplementary indicators of sustainability and quality of life
Healthy Cities Project Indicators [81]	WHO European Office initiated project that has helped to develop environmental health profiles of cities	Indicators include measures of health, health services, environment and socioeconomic aspects

This table is based on one originally developed by Lawrence [67]

The compilation of environmental urban indicators has many features in common with the corresponding health indicators. There are many variations (Indicators) on several themes (Domains). A host of individual indicators have been considered, but many of them are potentially interchangeable. Little empirical information, however, is available on their co-variation or their exchangeability. The exact balance of environmental indicators, social and economic determinants, and health outcomes in creating Indices is still an open issue though there appears to be general agreement that all should be part of such an Index.

#### Looking ahead—geospatial measurement of health and health disparity

A complementary approach to the assessment of health disparities is the burgeoning field of geovisualization. The growing armamentarium of data and geographic tools has given rise to alternate methods for measuring disparities, some of which can be married to the just described indicators and indices. For example, measures of urban design (enclosure, scale, transparency, complexity) can be obtained directly from digital sources and used to define urban space that may house the disadvantaged [68]. Remote sensing has been coupled with GIS methods (in Bangladesh, for example [69]) to demonstrate concentrations of poverty and the heterogeneity within impoverished areas. Techniques for assessing access to parks and other environmental landmarks have been used to provide measures of the availability of health activities within an urban space [70]. Google StreetView makes possible measures of local food availability with easy connection to population density and other factors that may affect disparities [71].

A series of studies from Australia demonstrate the potential melding of health and environmental indicators and geovisualization. Badland and colleagues identified 11 domains for “liveability” that included 61 usable indicators, and developed a framework that connected these indicators to social determinants of health [72]. They applied this concept to demonstrate the connection between Public Open Spaces (POS) and the mechanisms by which they influence health [73]. Similarly, a set of public transport indicators were developed, and their pathways of connection to population health explored [74]. This work-in-progress promises to bring environmental factors (open spaces, transport) together with health determinants in real space, and to serve as a complex metric for identifying health disparities.

## Conclusions

Despite the plethora of domains and indicators there are substantial commonalities among the major projects that have attempted to characterize health and disparities. These domains deal largely with health, irrespective of geo-location, and are usually at the regional or national level. Those that focus on urban issues often include environmental markers that affect health as well. The commonalities suggest that investigators share a common set of priorities but differ over the available welter of detail. An important area for further investigation is to explore that common ground, and determine—empirically, if not theoretically—the extent of correlation and exchangeability among indicators. Local urban areas would then have flexibility in the formation of indices based on locally available data.

There are commonalties among the approaches to Index formation as well. Several techniques for amalgamation of indicators are available, from simple linear combination to more sophisticated mathematical transformations and combinations. Many of these methods are transparent, and would be available to practitioners at the local level as well.

Measures that use indicators and indices to demonstrate disparities have been more elusive. Though considerable statistical development has gone into measures of disparity (see Table 1), those measures are largely a calculation created after the fact. (An example of an exception would be the inequality-weighted Human Development Index, [47] a valid and sophisticated measure, but one whose complexity hides raw differences.) The issue of demonstrating disparity re-invokes the question of scale. When applied globally, the disparity implicit in rank ordering of nations simply reports the difference between rich and poor. Attention to the detail within such ranking ignores Bryant’s admonition from 50 years ago. It ignores, as well, the spectrum of data and approaches required by the continuum from affluence to indigence. Issues of consummate importance for the latter (environmental quality, resource availability, public services, basic sanitation) have less immediacy for developed urban areas, though the microenvironment of some presumably affluent urban areas may well be substantially disadvantaged. Perhaps the real power of Indicators and Indices is to demonstrate disparity on the local level—a place where significant change may be possible. Locally collected data and simple, flexible tools for amalgamation, rather than fixed packages, may be a fruitful approach to understanding health disparity.

## Abbreviations

CEROI: Cities environment reports on the internet
GNP: Gross National Product
HDI: Human development index
HEART: Health equity assessment and response tool
UNDP: United Nations Development Program.
